# Azoxystrobin Impairs Neuronal Migration and Induces ROS Dependent Apoptosis in Cortical Neurons

**DOI:** 10.3390/ijms222212495

**Published:** 2021-11-19

**Authors:** Jieun Kang, Kausik Bishayee, Sung-Oh Huh

**Affiliations:** Department of Pharmacology, College of Medicine, Institute of Natural Medicine, Hallym University, Chuncheon 24252, Korea; wldms3313@gmail.com (J.K.); kausikbishayee1@gmail.com (K.B.)

**Keywords:** azoxystrobin, neurotoxicity, *in utero* electroporation, primary cortical neuron, mTORC1 activity

## Abstract

Fungicides often cause genotoxic stress and neurodevelopmental disorders such as autism (ASD). Fungicide-azoxystrobin (AZOX) showed acute and chronic toxicity to various organisms, and remained a concern for ill effects in developing neurons. We evaluated the neurotoxicity of AZOX in developing mouse brains, and observed prenatal exposure to AZOX reduced neuronal viability, neurite outgrowth, and cortical migration process in developing brains. The 50% inhibitory concentration (IC50) of AZOX for acute (24 h) and chronic (7 days) exposures were 30 and 10 μM, respectively. Loss in viability was due to the accumulation of reactive oxygen species (ROS), and inhibited neurite outgrowth was due to the deactivation of mTORC1 kinase activity. Pretreatment with ROS scavenger- N-acetylcysteine (NAC) reserved the viability loss and forced activation of mTORC1 kinase revived the neurite outgrowth in AZOX treated neurons. Intra-amniotic injection of AZOX coupled with *in utero* electroporation of GFP-labelled plasmid in E15.5 mouse was performed and 20 mg/kg AZOX inhibited radial neuronal migration. Moreover, the accumulation of mitochondria was significantly reduced in AZOX treated primary neurons, indicative of mitochondrial deactivation and induction of apoptosis, which was quantified by Bcl2/Bax ratio and caspase 3 cleavage assay. This study elucidated the neurotoxicity of AZOX and explained the possible cure from it.

## 1. Introduction

Azoxystrobin (AZOX) is a common strobilurins group fungicide widely used in agriculture to protect crops from fungal diseases [1]. Mechanistically, AZOX targets mitochondria and inhibits the oxidative phosphorylation process [2]. Inhibition of oxidative phosphorylation reduces cellular energy production [3]. Thus, exposure to this compound can produce unwanted side effects; in particular, exposure in pregnant women can be fatal for unborn babies. There is no report available on the impact of AZOX on neuronal development and maturation process. In this study, we examined the ill-effect of AZOX in neuronal maturation and development *in vitro* and *in vivo* mouse models.

The complex process for neurodevelopment is orchestrated by a specific signaling process. The imbalance in energy production and mitochondrial health could be fatal for the neurons [4]. This can either induce premature cell death or cell senescence, thus, as a consequence, the neurodevelopment process could be hampered [5]. The developing brain is extensively sensitive to neurotoxicants at levels far below those known to harm other organs in adults [6]. Neurotoxicity induced by environmental toxins can involve acute alterations in consciousness, seizures, cerebral infarctions, paralysis, neuropathy, and ototoxicity [7]. Previously it was shown that gestational exposure to fungicide Imidacloprid induced developmental delay, intellectual deficits, and mental disorders such as autism in children. Moreover, fungicides, such as pyraclostrobin, trifloxystrobin, famoxadone, and fenamidone have been linked to autism and neurodegeneration risk [8,9,10]. 

Fungicides, in many cases, are described as metabolic inhibitors and effectively suppress the metabolic capacity and ATP production of mitochondria. AZOX inhibits the ubiquinol (Qo) center of fungal respiration complex III through the cytochrome pathway [11]. Their non-targeted effects are often observed in higher mammals, including neonates and children. A previous study indicates that AZOX exposure altered lipid and glucose metabolism in liver cells [12], and affected the AMP-activated protein kinase (AMPK) pathway. Moreover, it induces mitochondria-mediated apoptosis in KYSE-150- esophageal squamous carcinoma cells [2]. In another instance, this fungicide was defined as a genotoxic element that alters the physiological process by oxidative damage at high concentrations, which can promote possible long-term biological consequences [13]. In lower organisms, AZOX compromises with superoxide dismutase (SOD), peroxidase (POD), glutathione S transferase (GST), glutathione peroxidase (GPx) activities, and glutathione (GSH) content, and induces reactive oxygen species (ROS) overproduction [14], demonstrated to cause acute mortality to tadpoles [15]. Due to the overwhelming off-target toxicity reports for AZOX, here, we demonstrated the possible molecular reason for toxicity in pregnant mice and its effect on the neurodevelopment process. 

Apoptosis induction was observed in AZOX treated cancer cells by depolarizing mitochondrial membrane potential [2] and enabled ROS pathways for cell death induction [16]. The integrative role of the molecular signaling pathway for toxicity was not thoroughly studied for AZOX. In this research, we delineated a molecular mechanism for AZOX mediated cytotoxicity against developing neurons. The mTOR has a distinct role in controlling protein synthesis, metabolism, lysosomal accumulation, and mitochondrial functions, and is likely to affect cellular health [17,18]. The mitochondrial function and biogenesis process is managed by mTORC1 through translating their genes [19], and an increase in mTOR activity promotes axonal growth and development in embryos [20]. Different toxicants weaken the cells by lowering their mTOR activity, which affects vast downstream activities; as such, different stress and cell death-related cascades are activated in low mTOR conditions.

Here, we examined the toxicity of AZOX in developing mouse embryos. In brief, we observed the cortical brain development was impaired in AZOX treated groups due to inhibited neuronal migration and primary neurite formation process. This, in turn, could lead to the development of symptoms characteristic of psychiatric disorders such as autism. Moreover, interneuron migration controls cerebral cortical growth, and its impairment could lead to macrocephaly [21]. Neurite maturation and viability diminished by AZOX were dependent on mTORC1 activity. Either forced activation of mTORC1 or N-acetylcysteine (NAC) treatment ameliorated neurite defects and cell viability loss in primary cortical neurons caused by AZOX.

## 2. Results

### 2.1. AZOX Reduced Viability in Primary Cortical Neuron Cultures by Elevating ROS Level 

**Figure 1:**
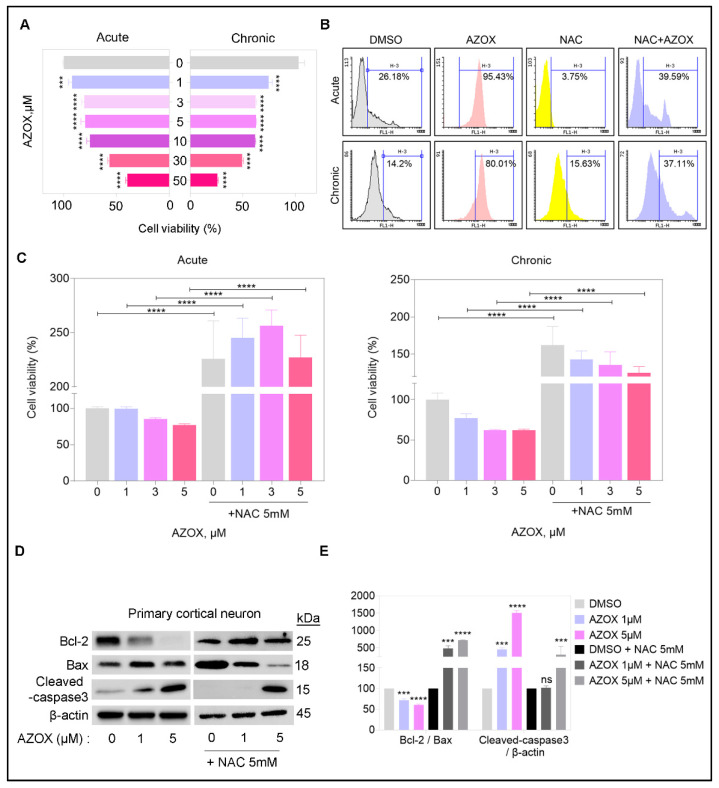
Cytotoxicity of AZOX in primary cortical neurons (**A**) AZOX was exposed to primary cortical neuronal cultures (stage E15.5) for a short interval (acute-24 h) and a long interval (chronic-7 days). Cell viability assay was performed in primary neurons exposed to different concentrations of AZOX. The 50% inhibitory concentration (IC50) for AZOX was 30 μM for acute treatment, and 10 μM for chronic exposure for 24 h (acute exposure). (**B**) ROS generation in the primary neurons, quantified by oxidized DCFDA by flow cytometry. (**C**) Pre-exposure of NAC revived the viability in AZOX treated primary neurons. (**D**,**E**) Quantification of the protein expression of apoptotic related markers Bcl-2/Bax ratio and cleaved caspase 3 in AZOX and AZOX + NAC treated primary neurons. The histograms represent the band intensity, data are presented as mean ± SEM; *t*-test: *** *p* < 0.001, **** *p* < 0.0001. β-actin was used as the loading control.

Previously, AZOX cytotoxicity was shown in different cell lines by ROS induction. Here, we examined the acute and chronic AZOX effect on primary cortical cultures. AZOX was exposed to primary cortical neuronal cultures (stage E15.5) for a short interval (acute-24 h) and a long interval (chronic-7 days). We observed a dose-dependent decrease in viability of primary neuronal cells (Figure 1A). The chronic exposure to AZOX had higher toxicity in terms of viability loss. The 50% inhibitory concentration (IC50) for AZOX was 30 μM for acute treatment, and 10 μM for chronic exposure.

We found that 5 μM AZOX significantly increased ROS generation in the primary neuronal cells, quantified by oxidized DCFDA flow cytometry staining (Figure 1B). The ROS accumulation in the primary neurons was significantly higher in the presence of AZOX in both acute and chronic exposure. Preexposure of NAC significantly reduced cellular ROS levels in both vehicles and AZOX treated primary neurons (Figure 1B). We observed NAC treatment itself increased the viability in primary neurons comparing vehicle control (Figure 1C), implying the potential of NAC to inhibit cell death in neurons [22]. Similarly, apoptotic cell death was decreased in cultures with NAC treatment, compared with that of cells treated with vehicle only (Figure 1D,E). While comparing the general viability changes, NAC was effective against AZOX, but there was a significant loss in viability in the AZOX-NAC sets. The pretreatment of ROS scavenger NAC reduced the AZOX cytotoxicity in primary neurons in terms of viability loss in acute and chronic treatment (Figure 1C). To check the cell death and apoptosis caused by AZOX, we quantified the protein expression of apoptotic related markers Bcl-2/Bax ratio and cleaved caspase 3. The apoptosis markers were elevated in AZOX treated neurons, while pre-exposure to NAC markedly reduced the apoptosis markers (Figure 1D,E), more strikingly in 1 μM AZOX treated neurons. We observed caspase 3 activity persisted in NAC-AZOX (5 μM) neurons, therefore the reduction in viability might be the effect of this. Taken together, the cytotoxicity of AZOX was due to ROS accumulation, and pre-exposure of a ROS scavenger NAC was able to reduce the viability loss and apoptotic cell death in primary neurons. Thus, ROS scavenger treatment has significantly improved the condition in primary neurons, and protects them from death by apoptosis. 

### 2.2. AZOX Regulated Mitochondrial Mass and Polarization (MMP) and Activated Caspase 3 in Primary Neurons

**Figure 2:**
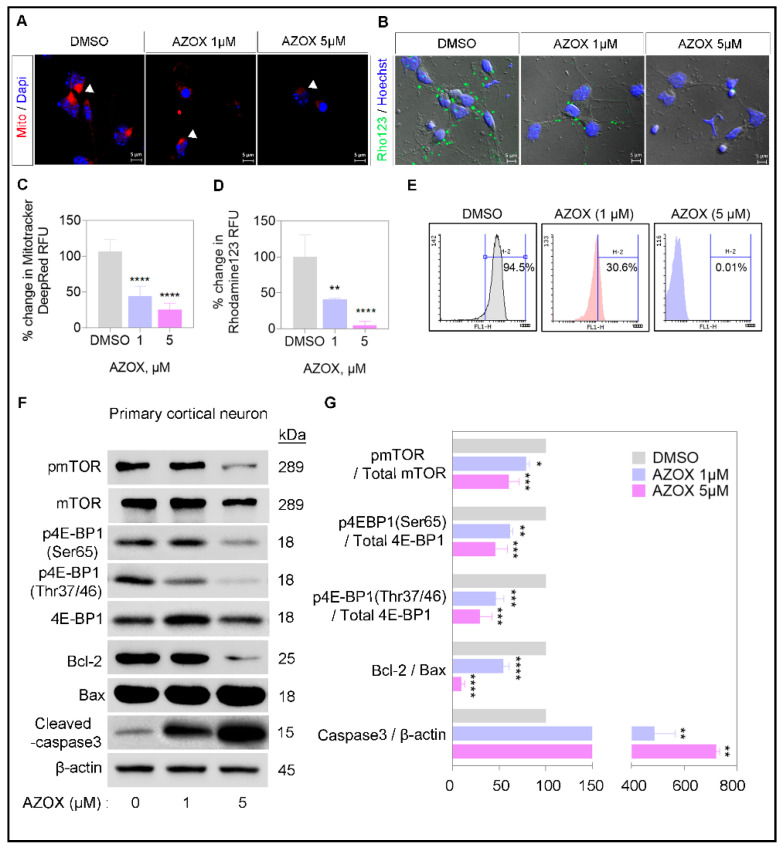
AZOX depolarizes the mitochondria membrane. (**A**–**D**) Mitochondria accumulation and MMP were examined by MitoTracker Deep Red and Rho123 staining in primary neurons, respectively. Fluorescence intensity was quantified using ImageJ software and plotted as a histogram. Scale bar corresponds to 5 μm, data are presented as mean ± SEM; *t*-test: * *p* < 0.05, ** *p* < 0.01, *** *p* < 0.001, **** *p* < 0.0001. (**E**) Flow cytometry analysis of primary neurons stained with Rho123. (**F**,**G**) mTORC1 activity in the primary cortical neuron (DIV3) was quantified by Western blot, and the band densitometry was measured using ImageJ software. The histograms represent the band intensity, data are presented as mean ± SEM; *t*-test: * *p* < 0.05, ** *p* < 0.01, *** *p* < 0.001, **** *p* < 0.0001. β-actin was used as the loading control.

Mitochondrial viability and accumulation is an essential aspect for maintaining cellular growth. The viability of the cells reduces when mitochondria become damaged. To observe mitochondrial mass and membrane potential (Δψ) in neurons, we stained the cells with a mitochondria-specific dye, MitoTracker and Rho123 in AZOX or DMSO treated primary neurons, respectively. MitoTracker readily stained the mitochondria, and was found in an accumulated state in control experiments. In AZOX treated cells, the intensity of mitochondria staining was significantly reduced (Figure 2A,B), determining the reduction in mitochondrial mass. Moreover, Rho123 staining, quantified by flow cytometry and confocal microscopy, was significantly decreased in AZOX treated cells, implying the depolarization of the mitochondrial membrane potential. Consequently, we observed the decrease in Bcl-2/Bax ratio and induction in apoptosis marker, caspase 3 cleavage, in AZOX treated neurons (Figure 2C,D). Thus, the cell death induction in AZOX cells was apoptotic.

In previous studies, we found that mTORC1 kinase was a regulator of mitochondrial activity and the biogenesis process [18]. mTORC1 excel in the transcription and translation of mitochondria biogenesis genes via 4E-BP1 phosphorylation [23]. Phosphorylation of mTOR (Ser2448) and 4E-BP1 (Ser46 and Thr37/46) was reduced in AZOX treated primary neurons (Figure 2C,D), implying the reduction in the mitochondrial biogenesis process. AZOX mediated toxicity was due to damages in mitochondria, which potentiated apoptosis induction. Moreover, AZOX treatment deactivated mTOR and downstream 4E-BP1, which is known for feeding into the mitochondrial biogenesis process. 

### 2.3. AZOX Impaired Radial Migration in Neo-Cortex

A neuronal migration process is required during neo-cortex development for proper alignment and distribution of newborn neurons. Neuronal migration is orchestrated by multigene coordination, and critically regulated during neo-cortex development [24]. Any irregularity can impair the process of migration, and lead to the induction of development disorders like ASD [25]. Previously, we observed AZOX treatment reduced mTORC1 activity by suppressing the mTOR phosphorylation (Figure 2C). The mTOR activity is finely tuned for neuronal development; any irregularities in mTOR signaling by silencing or mutations can disrupt the neo-cortex migration process [26,27]. 

**Figure 3:**
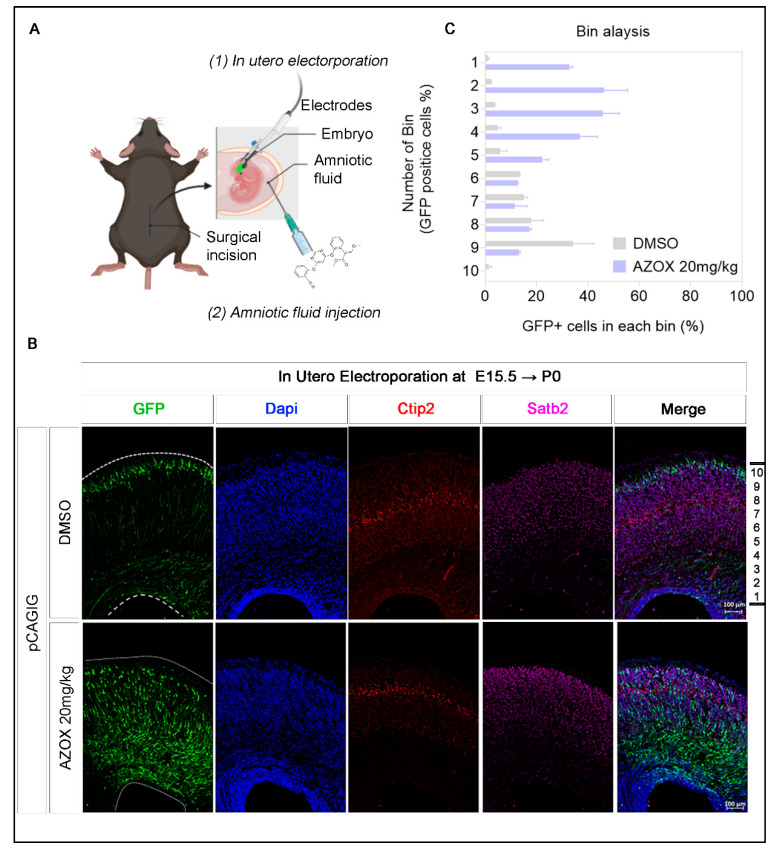
AZOX inhibits radial migration in developing mouse brains. (**A**) Schematic for microinjection procedure in embryonic mouse brain (diagram created with BioRender.com). (**B**,**C**) Coronal sections of mice telencephalon at P0 stage after *in utero* electroporation at E15.5. Radial migration was delayed in AZOX exposed brains. Quantitative layer marker (Ctip2, Satb2) showing the distribution of transfected neurons across the cortex, scale bar corresponds to 100 μm. The neuronal distribution in the cortex was quantified by Bin analysis.

In this section, we examined the effect of AZOX in neo-cortex development and migration. Here we developed a co-injection technique for AZOX treatment and neuronal tracing with intra-amniotic AZOX micro-injection and labeling neurons with GFP-plasmid (pCAGIG vector) (Figure 3A). We micro-injected AZOX (20 mg/kg) and DMSO into the amniotic fluid, along with GFP labeled vector (to trace neurons) in the ventricular zone of E15.5 embryos via *in utero* electroporation. We harvested the embryos at P0, and observed that most of the neurons in control were migrated to the cortical plate (CP), and neurons with AZOX remained either in the intermediate zone (IZ) or in the ventricular and sub-ventricular zone (VZ/SVZ) (Figure 3B). To quantify the distribution of neurons in the cortex, we analyzed the neuronal migration at P0 after dividing the cortical layer into 10 bins (Figure 3B). We found the neurons in control sets were distributed intensively in bin numbers 9 and 10 that correspond to the upper layer of the cortex.

In contrast, neurons were primarily distributed in bins 2 and 4 in AZOX injected brains, which corresponds to the IZ of the cortex (Figure 3C). Therefore, AZOX injected embryos displayed a significant delay in migration, in comparison with the control. Of note, the conventional thickness and distribution of the layer V-VI marker, Ctip2 was significantly reduced in AZOX treatment. Therefore, we found that AZOX impaired the neo-cortical migration process and cortical neuron distribution in developing mouse brains.

### 2.4. AZOX Inhibited Neurite Formation, Axons Branching, and Elongation in Primary Neurons

**Figure 4:**
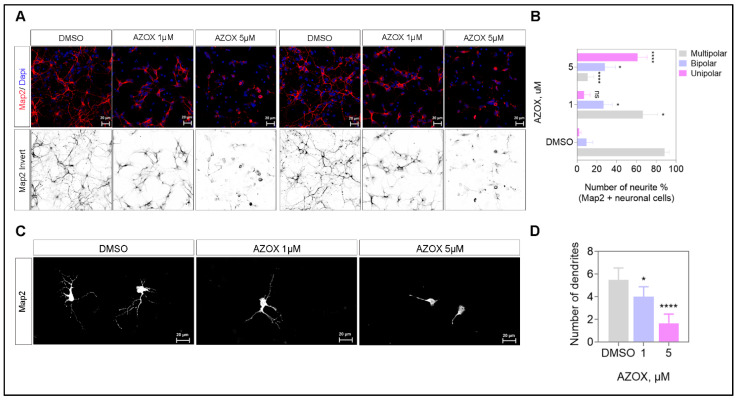
AZOX inhibits neurite formation in primary neurons. (**A**,**B**) primary neurons were cultured and exposed to 1 and 5 μM AZOX for 24 h and stained with the Map2 (red), Dapi (blue), scale bar corresponds to 20 μm. Azoxystrobin 1 μM and 5 μM reduced the dendritic complexity. Histograms show quantification of dendritic complexity in primary neurons, data are presented as mean ± SEM. (**C**,**D**) Quantification of dendritic numbers in AZOX treated neurons, scale bar corresponds to 20 μm. The histogram represents the quantification of dendritic numbers in primary neurons; data are presented as mean ± SEM; *t*-test: * *p* < 0.05, **** *p* < 0.0001.

In the previous section, we observed that AZOX was responsible for inhibiting the neo-cortical migration process. One prominent mechanism for the defective migration process is deformed neurite formation in neuronal cells. Therefore, we examined neurite formation in mouse primary cortical neurons on AZOX exposure for 24 h. We treated AZOX and DMSO in primary neurons, and analyzed the dendritic complexity at three days *in vitro* (3 DIV). We conducted MAP2 (dendritic marker) staining with DAPI (nucleus) in primary cortical neurons (Figure 4A). We grouped the MAP2 positive neurons in three tires by their dendrite numbers, namely unipolar, bipolar, and multipolar. The multipolar was considered as the most complex over bipolar and unipolar neurons. Observed AZOX significantly reduced the multipolar neurons, and the effect was most prominent in 5 μM AZOX treated neurons, compared with the control. Moreover, we observed the number of unipolar neurons was significantly increased at the high dosed AZOX set. Thus, AZOX effectively reduced the dendritic complexity compared with DMSO exposed neurons (Figure 4B). In addition, dendrite numbers were reduced in AZOX treated neurons (Figure 4C,D). These results indicated that AZOX inhibited neurite formation. 

### 2.5. AZOX Regulated Neurite Outgrowth in mTORC1 Dependent Manner 

**Figure 5:**
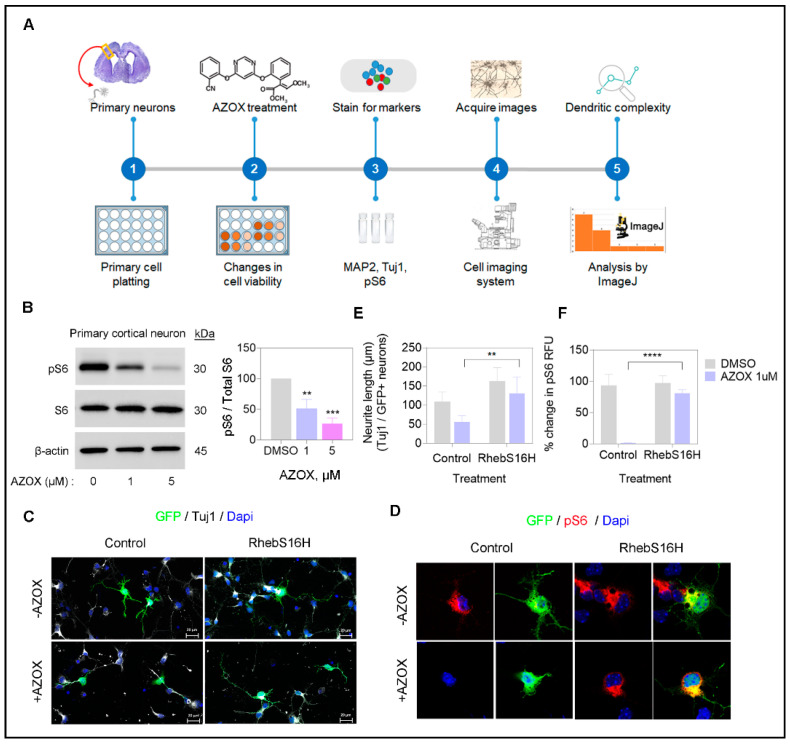
Forced activation of mTORC1 by RhebS16H overexpression rescues neurite loss in AZOX treatment. (**A**) Schematic for evaluating neuronal toxicity and quantification (**B**) pS6 expression level detected by Western blotting. The histogram represents the band intensity, data are presented as mean ± SEM; *t*-test: ** *p* < 0.01, *** *p* < 0.001, **** *p* < 0.0001. β-actin was used as the loading control. (**C**,**D**) The neurite length was quantified by immunostaining primary cortical neurons (3 DIV) with Tuj1. Neurons were IUE at E15.5 with a Rheb S16H construct and stained at 3 DIV with anti-GFP (green) and pS6 (red), Tuj1 (Purple). Scale bar, 20 µm. (**E**,**F**) Histogram represents the longest neurite length (*t*-test: ** *p* < 0.01). RhebS16H transfected neurons showed longer axons in 1 µM AZOX treated neurons. The pS6 staining represented mTORC1 activation by Rheb S16H overexpression. Scale bar corresponds to 20 μm and data are presented as mean ± SEM; *t*-test: ** *p* < 0.01, *** *p* < 0.001, **** *p* < 0.0001.

Neurons form axons and dendrites as their cellular body extensions, also referred to as neurites. These neurite outgrowths are regulated via complex intracellular signaling events [28]. Neurite outgrowth is a commonly used assay for studying neuronal development and neuronal degeneration in primary cortical neurons. The neurite outgrowth can be inhibited by various neurotoxic chemicals. We have defined the toxicity generated by AZOX in primary neurons isolated from the E15.5 mouse brain (Figure 5A). The AZOX treated neurons showed a reduced pS6 expression (Figure 5B), thereby implying the reduction in mTORC1 activity. 

Mounting evidence suggests that activated mTORC1 and its downstream S6K enhance axon formation and elongation [13]. Here, we investigated whether the reduction of neurite formation on primary cortical neurons by AZOX treatment was related to the mTORC1-S6 axis. We observed markers Tuj1 and pS6 were significantly reduced in AZOX treatment, implying a reduction in neurite length and S6 activity (Figure 5C–F). 

Additionally, we performed an addback experiment that showed forced activation of mTORC1 by RhebS16H overexpression significantly reduced the AZOX toxicity. The axon lengths were measured after three DIV for the treated cells. We performed immunocytochemistry using tuj1 as a neuronal/axonal marker. Tuj1 staining showed the neurite length was significantly decreased in AZOX treatment sets (56 ± 3.5 μm), along with a decrease in pS6 expression (Figure 5C–F) compared with the control vector (109 ± 5.2 μm). In contrast, in AZOX with RhebS16H overexpression (131 ± 9.2 μm) promoted axonal growth, and increased pS6 expression. The forced activation of the mTORC1-S6K axis by RhebS16H overexpression was essential for reducing the toxic effect of AZOX on neurite formation.

## 3. Discussion

We described the potential neurotoxic nature of AZOX in mouse embryos. The acute and chronic exposure of AZOX was cytotoxic to the primary cortical neuron culture. The viability loss due to AZOX was ameliorated by NAC pre-exposure in the neurons (Figure 1). We also observed that AZOX exposure reduced the primary neurite length while losing their viability. The length reduction of the neurite was fixed by forced activation of mTORC1 kinase by transfecting active Rheb S16H in the neurons via *in utero* electroporation in the embryonic mouse brain (Figure 5). The previously cytotoxic nature of AZOX was described against different cancer cells and, based on the evidence, several anti-cancer drugs show significant neurotoxicity in clinics [7]. Thus we assumed AZOX may show neurotoxicity in neuronal cultures and corticogenesis. The dendrite marker MAP2 acts as a sensor for neurotoxicity in CNS neurons [7]. We observed reduced MAP2 staining in AZOX treated neurons, indicating toxicity and decreases in neuronal dendrite counts and length (Figure 4). 

**Figure 6:**
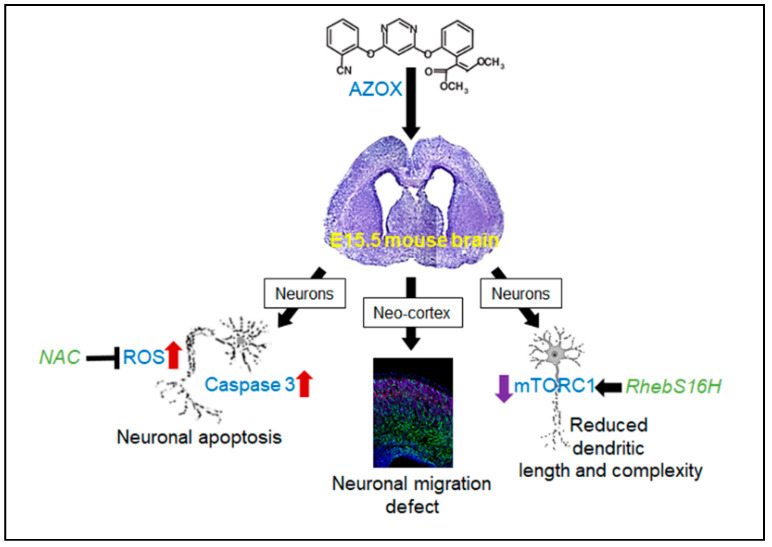
AZOX produced neurotoxicity and reduced neurite formation, and that was dependent on mTORC1 activity. Induced mTORC1 activity prevented AZOX effect on neurite formation; ROS was accumulated in neurons and caused viability reduction, NAC treatment reduced ROS mediated cell death and apoptosis in primary neurons. The axonal and dendritic branching was reduced in AZOX treated neurons, which was improved by the forced activation of mTORC1 by RhebS16H. Lastly, AZOX has inhibited neuronal migration in the neo-cortex.

The primary mechanism involved in the shortening of dendritic length and complexity was the deactivation of mTORC1 activity. Our observations of changes to cellular morphology could be controlled by forced activation of mTORC1 by Rheb S16H overexpression (Figure 5). In most cells, mTORC1 regulates protein translation by controlling ribosome assembly and translation initiation [29]. The changes in phosphorylation of mTORC1 downstream by AZOX determined the involvement of translation and protein synthesis regulators 4EBP1 and S6, respectively. The phosphorylation of 4EBP1, S6, and mTOR were inhibited by AZOX (Figure 2 and Figure 5), which could downregulate protein synthesis, mitochondrial biogenesis, and respiration or metabolism (Figure 6). Alternatively, mTORC1 may indirectly affect cytoskeletal proteins needed for axon-dendrite formation and maintenance [28], suggesting that the dendritic morphology and formation process may represent a crucial regulatory hub for cortical maturation. 

Abnormalities in mTOR activity are linked with severe nervous system development deficits, including tumors, autism, and seizures [22]. The metabolic imbalance created due to irregularities in mTOR activity affects mitochondrial morphology and integrity [30]. The mTORC1/4E-BP1 pathway promotes oxidative phosphorylation in mitochondria. Thus, mTORC1 drives growth by simultaneously activating the translation of mRNAs that encode proteins involved in cellular energy production [21]. We observed AZOX regulated the metabolic capacity of mitochondria, along with inhibition in mTOR and 4EBP1 phosphorylation (Figure 2). This may degenerate the efficiency of the transcription process of mitochondrial biogenesis genes in the nucleus.

AZOX, which is a natural product of the Strobilurins group of compounds, works by binding to cytochrome b of complex III (CIII) of the mitochondria, and disrupts mitochondrial oxygen consumption [31]. The tissue distribution of AZOX was predicted previously by ADMET software, and was positive for intestine absorption, and permeable for the blood-brain barrier and epithelial cells (Caco2) [32]; the presence of radiolabeled AZOX was found in rat brain when seven days’ dosing of 1 mg/kg AZOX was administrated [33]. Energy deficiency in mitochondria induces mitochondrial fission. Dysfunctions of mitochondrial dynamics contribute to several inherited and age-associated neurodegenerative diseases [34]. ROS triggers mitochondrial permeability transition pore (mPTP) induction within individual mitochondria in intact cell systems [35]. The massive release of mPTP certainly depolarizes the mitochondria’s inner membrane potential. 

MitoTracker staining in AZOX-treated neurons showed a less staining capacity in mitochondria (Figure 2). MitoTracker probes contain a mildly thiol-reactive chloromethyl moiety that accumulates in active mitochondria. The dye is readily sequestered by functioning mitochondria; these stains are easily washed out of cells once the mitochondria experience a loss in membrane potential. The high ROS level activates mitochondria-mediated apoptosis pathways that are capable of inducing cell death [36]. AZOX caused Bcl2/Bax-related intrinsic mitochondrial pathway of apoptosis and cleaved caspase 3 (Figure 1 and Figure 2) to induce apoptosis and cell death. Implementing ROS scavenger- NAC on top of AZOX relieves the neuronal cells from reducing viability (Figure 1). NAC is a heavily applied antioxidant *in vivo* and *in vitro* as a nutritional supplement. It can restrict the apoptotic pathway through the inhibition of caspase-activity, and as a precursor to L-cysteine that triggers glutathione-elevated biosynthesis. It acts directly as a scavenger of free radicals, especially oxygen radicals [37]. Reduced mTOR and induced apoptosis in AZOX treated neurons may inhibit many important signaling cascades that promote the cortical migration process [38]. The cortical migration process in the developing brain was significantly reduced by AZOX treatment, indicating defects in neuronal development in mouse embryos (Figure 3). 

In summary, AZOX modulated mTORC1 activity and generated ROS (Figure 1 and Figure 2). The ROS-mitochondria pathway was predictive for neuronal viability loss (Figure 1), while mTORC1 was the primary mechanism involved in the shortening of dendritic length and complexity in primary neurons (Figure 4). Moreover, we showed that activation of mTORC1 compensated the dendritic length and complexity, and reduction in ROS made the neurons viable (Figure 6). Therefore, our findings demonstrated the potential efficacy of the mTORC1 pathway as a therapeutic target to enhance regeneration after neurotoxic exposure.

## 4. Materials and Methods

### 4.1. Experimental Animals and Isolation of Primary Neurons

The experiments were conducted using pregnant C57BL/6J mice purchased from Daehan Bio-Link (Eumseong-gun, South Korea). The animals were housed at optimal conditions (12 h light and dark cycles at 22 ± 2 °C, 50 ± 10% humidity), and their food was procured from a commercial food company- Purina Inc. (Sungnam si, South Korea). The experiments were performed in compliance with the proper animal handling guidelines established by the Institutional Animal Care and Use Committee (IACUC) of Hallym University (ethical clearance number: Hallym2019-36, South Korea). 

Primary cortical neurons were isolated from the embryos at the E15.5 stage. In brief, the brain cortex was isolated, and the tissue fragments were digested in TrypLE^TM^ (Gibco, Waltham, MA, USA) supplemented with DNase I (100 μg/mL) (Sigma, St. Louis, MO, USA). The digested cells were then washed in 1X HBSS (Gibco) at room temperature. The isolated cells were then cultured in selective media.

### 4.2. Primary Neuron Cultures

Primary isolated neurons were cultured in a neurobasal medium (Invitrogen, Waltham, MA, USA) supplemented with 2% B-27 (ThermoFisher, Waltham, MA, USA) and antibiotics. The cortical neurons were plated either on coverslips or on cell culture plates coated with poly-L-lysine (Sigma). The culture media was replaced with fresh media after 2 h of cell seeding, and cultures were incubated in a 5% CO_2_ incubator at 37 °C. 

### 4.3. Azoxystrobin Treatment and Cell Viability Assay

Treatments stock solutions of AZOX (FUJIFILM, Tokyo, Japan) were diluted in DMSO (Sigma), and then stored at −80 °C. Neurons were exposed to AZOX at 1 DIV for seven days for chronic exposure experiments, and 24 h in acute exposure experiments.

Primary neurons were plated in a 96-well plate at a density of 3 × 10^4^ cells/well. After AZOX treatment, the viability of the primary neurons was tested using Quanti-MAX WST-8 Cell viability assay kit (BIOMAX, Seoul, South Korea). Fluorescence was measured at 450 nm, and viability histograms were plotted using GraphPad Prism 8 software (GraphPad, San Diego, CA, USA).

### 4.4. Identification of ROS Using Oxidized DCFDA and Flowcytometry

We used cell-permeable fluorescent probes 2′,7′-dichlorodihydrofluorescein diacetate (DCFDA; D6883; Sigma) to measure the cellular redox state of AZOX or DMSO treated primary cells. 2′,7′-dichlorofluorescin diacetate is a widely used technique for directly measuring the redox state of cells. 2′,7′-dichlorofluorescin diacetate is a cell-permeable non-fluorescent probe. 2′,7′-dichlorofluorescin diacetate is de-esterified intracellularly and turns to highly fluorescent 2′,7′-dichlorofluorescein upon oxidation. Cells were harvested and stained with DCFDA solution (1 μg/mL) in PBS for 30 min in the dark. The stained cells were washed in ice-cold PBS two times, and fluorescence intensity was measured by flow cytometry at the FL1-H filter.

### 4.5. Preparation of Plasmids Constructs and In Utero Electroporation 

Active Rheb construct was made by cloning Rheb (S16H) (GenBank sequence FQ219039.1) in pCAGIG vector (plasmid 11159, Addgene, Watertown, MA, USA), and an empty pCAGIG vector served as a control. 

As described in the previously published protocol, the *in utero* electroporation (IUE) was performed accordingly [39]. Pregnant mice were anesthetized with isoflurane at E15.5, and laparotomy was performed to expose the cervix. The endotoxin-free DNA plasmid vectors (1 μg/μL) were prepared in phosphate buffer saline (PBS) containing 0.01% Fast Green dye (Sigma) solution. The DNA solution was injected into the lateral ventricles of E15.5 embryos using microinjection with a sterilized pulled glass micropipette. After DNA microinjection, the embryos were electroporated using 5 nm diameter Tweezertrodes (Harvard Bioscience, Holliston, MA, USA). The electroporation was applied using a square-wave pulse generator (ECM 830; Harvard Bioscience) by five 40 V square pulses of 50-ms with 950 ms intervals. The uterine horns were then returned to the abdominal cavity, allowing the embryos to continue their development inside the mother’s womb.

### 4.6. Amniotic-Fluid Injection

To identify AZOX’s effect on mouse brain development, we anesthetized pregnant C57BL/6J mice on E15.5 with continuous isoflurane. We performed intrauterine injections of AZOX or DMSO mixed in phosphate-buffered saline solution (PBS) using a Hamilton syringe (0.05 mL), intra-amniotic injection with DMSO, or AZOX in PBS nto each gestational sac.

### 4.7. SDS-PAGE and Immunoblots 

Primary cortical neurons were lysed using Radio-immunoprecipitation assay buffer (RIPA) buffer (50 mM Tris-HCl, pH 7.5, 150 mM NaCl, 1 mM EGTA, 1 mM EDTA, 1% Triton X-100, 1 mM Na3VO4, 5 mM NaF, and a protease inhibitor cocktail). Lysates were centrifuged at 15,000× *g* for 20 min at 4 °C, and the supernatant was transferred to fresh tubes. The obtained protein was measured by a BCA assay kit (Bio-Rad, Hercules, CA, USA). 

Equal amounts of protein were heated in a 4X protein-loading buffer, and were subjected to SDS-PAGE for protein size separation. After gel electrophoresis, proteins were transferred to polyvinylidene difluoride (PVDF) membranes (Millipore, Burlington, MA, USA) and blocked in 5% skimmed milk in 1X TBST for 1 h. The membranes were probed in primary antibody for overnight at 4 °C and washed three times with 1X TBST (1X TBS, 0.1% Tween-20), and then incubated in appropriate secondary antibodies. After washing three times in 1X TBST, the protein bands were detected using Western HRP substrate (Luminata Forte, Millipore) in a chemiluminescence imaging system (Fusion FX, Vilber Lourmat, Collégien, France). The antibodies used are listed in Table 1.

### 4.8. Immunohistochemistry and Immunocytochemistry

The IUE brains were collected and fixed in 4% paraformaldehyde at 4 °C for overnight, and placed in 30% sucrose PBS solution at 4 °C overnight, then embedded in optimal cutting temperature (OCT) compound. All brain samples were sectioned at a coronal angle of 10 μm thickness using a cryo-section machine. The sectioned samples were blocked in 3% bovine serum albumin (BSA) in PBS solution containing 0.1% Triton X-100. After blocking for 1 h, they were incubated in primary antibodies at 4 °C for overnight. Next, samples were washed three times with 1XPBS and incubated in Alexa Fluor^®^ conjugated secondary antibody (Life Technologies, Carlsbad, CA, USA) at 1:1000 dilution at room temperature in the dark for 2 h. The stained samples were mounted in mounting media and photographed using a confocal microscope (LSM710, Carl Zeiss, Oberkochen, Germany). The antibodies used for IHC are listed in Table 1. 

For immunocytochemistry (ICC), the primary neurons were cultured, then fixed and permeabilized. After washing, cells were blocked by 3% BSA in PBS solution containing 0.1% Triton X-100 at room temperature for 1 h. The cells were incubated in primary antibodies at 4 °C, overnight and then in Alexa Fluor^®^ conjugated secondary antibodies (Life Technologies) for 2 h at room temperature in the dark. Finally, the samples were mounted in mounting media and photographed using a confocal microscope (Carl Zeiss). The antibodies used for ICC are listed in Table 1.

### 4.9. Neurite Outgrowth Assay 

Primary cortical neurons were cultured in a neurobasal medium without or with AZOX for 24 h, and then immunocytochemistry was performed with the anti-TuJ1 antibody at 3 DIV. The neurite length (the length of the longest neurite in TuJ1-positive neuron) per neuron was measured using ImageJ software (NIH, Bethesda, MD, USA).

### 4.10. Mitochondria Staining and Membrane Potential

Neurons were treated with either DMSO or AZOX doses and, the cells were stained by either MitoTracker or Rho123 for evaluating mitochondrial mass and membrane potential, respectively. Mitochondrial mass was stained using 500 nM MitoTracker Deep Red FM (M22426; Invitrogen), diluted in the culture media, and incubated for 45 min at 37 °C in a CO_2_ incubator, then subsequently fixed in 4% PFA, washed 3 times in PBST, and counterstained with DAPI. Mitochondrial membrane potential (Δψ) changes have been evaluated by measuring Rhodamine 123 (Rho123; R8004; Sigma) fluorescence quenching by flow cytometry and confocal microscopy. Cells were incubated in 10 µM Rho123 for 20 min after fixation, and the nucleus was counterstained by Hoechst 123 (H3570; Invitrogen). The samples were washed three times in PBS before mounting, and the photographs were acquired using a confocal microscope.

### 4.11. Statistical Analysis 

Statistical analysis was performed by using GraphPad Prism8 software (GraphPad, San Diego, CA, USA). The *p*-values were calculated from one-way ANOVA analysis with a post-hoc Tukey test. Data were presented as mean ± SEM; * *p* < 0.05, ** *p* < 0.01, *** *p* < 0.001, **** *p* < 0.0001 were considered significant. All the experiments were performed multiple times for reproducibility. 

## Figures and Tables

**Table 1:** List of antibodies used for Western blot (WB), immunocytochemistry (ICC), and immunohistochemistry (IHC).

No.	Antibody	Species	Applications	Dilutions	Manufacturer	Cat. No.
1	Phospho-mTOR (S2448)	Rabbit	WB	1:1000	Cell Signaling Technology	5536
2	mTOR	Rabbit	WB	1:1000	Cell Signaling Technology	2983
3	Phospho-4E-BP1 (Ser65)	Rabbit	WB	1:1000	Cell Signaling Technology	9451S
4	Phospho-4E-BP1 (Thr37/46)	Rabbit	WB	1:1000	Cell Signaling Technology	2855S
5	4E-BP1	Rabbit	WB	1:1000	Cell Signaling Technology	9452S
6	Phospho-S6	Rabbit	WB, ICC	1:1000	Cell Signaling Technology	5364
7	S6	Mouse	WB	1:1000	Cell Signaling Technology	2317S
8	Bcl-2	Rabbit	WB	1:1000	Cell Signaling Technology	2876
9	Bax	Rabbit	WB	1:1000	Cell Signaling Technology	2772
10	Caspase3	Rabbit	WB	1:1000	Abcam	ab4051
11	Map2	Rabbit	ICC	1:500	Abcam	ab32454
12	beta-3-tubulin (Tuj1)	Mouse	ICC	1:100	Cell Signaling Technology	4466
13	GFP	Chicken	ICC, IHC-IF	1:1000	Abcam	ab13970
14	Ctip2	Rat	IHC-IF	1:500	Abcam	ab18465
15	Satb2	Mouse	IHC-IF	1:500	Abcam	ab51502
16	β-actin	Rabbit	WB	1:1000	Cell Signaling Technology	4967

## Data Availability

Data is contained within the article.
